# Cultivating distress: cotton, caste and farmer suicides in India

**DOI:** 10.1080/13648470.2021.1993630

**Published:** 2021-11-03

**Authors:** Nanda Kishore Kannuri, Sushrut Jadhav

**Affiliations:** a Indian Institute of Public Health, Hyderabad, Public Health Foundation of India, Hyderabad, Telanga State, India; b Division of Psychiatry, University College London, London, UK.

**Keywords:** Farmer suicides, cotton, caste, distress, clinical anthropology, panlexicon

## Abstract

Nearly 4,00,000 farmers committed suicide in India between 1995 and 2018. This translates into approximately 48 suicides every day. The majority of suicides were those from ‘backwarded’ castes including Dalit farmers. This ethnographic study on cotton farmer suicide reports narratives of surviving Dalit families. The results reveal that financial and moral debt when accrued within a web of family and caste-related relationships result in patterns of personal and familial humiliation, producing a profound sense of hopelessness in the Self. This loss of hope and pervasive humiliation is ‘cultivated’  by a cascade of decisions taken by others with little or no responsibility to the farmers and the land they hope to cultivate as they follow different cultural and financial logic. Suicide resolves the farmers’ humiliation and is a logical conclusion to the farmer’s distress, which results from a reconfiguration of agricultural spaces into socially toxic places, in turn framing a local panopticon. The current corona virus pandemic is likely to impact adversely on peoples who are culturally distanced.

## Introduction

India is a largely rural, agricultural country. The latest census report suggests that 55% of the Indian population engage in Agriculture (Government of India 2011). The Green Revolution, spanning the 1960s and 70s, resulted in a drastic shift of agricultural practices in India. The technologies created across the two decades, backed by input subsidies, helped rich peasants gain wealth and political power at the expense of small and marginal farmers (Ghatak 2000). Farmer movements led by wealthy farmers joined the Green Revolution since the 1970s and also appealed to small and marginal farmers.^1^

Agricultural policies were also affected by a move towards liberalisation and globalisation during the early 1990s. As a result of this drive, national and state interventions favoured policies that prioritised the open market and were oriented around capital. This had a disproportionate and adverse effect on small and marginal farmers leading to the withdrawal of state subsidies with minimum support prices for procurement, and expose them to the volatility of global market (Ghosh 2005; Menon 2006). The open market also promoted the entry of Multi-National Companies (MNCs) into farming sector, ushering in an era of expensive hybrid seeds. Consequently, most small and marginal farmers, in order to make up for the escalating input costs of seeds, fertilisers and labour, shifted farming from food to commercial crops such as cotton, chillies and sugarcane (Ghatak 2000; Gupta 2021).

The national economic reforms of the 1990s included reduced public investment in agriculture, resulting in an agrarian crisis that led to an epidemic of farmer suicides (Ghosh 2005). The state withdrew funding from agricultural development in vital areas such as agricultural support services, which had previously allowed for the exchange of knowledge, facilities and research. This led to a decline in agricultural growth, and negatively affected the livelihoods of farmers and others who depended on sub-sectors of agriculture such as horticulture and livestock. Over a decade ago, India’s national agricultural policy formally recognised that agriculture had become ‘a relatively unrewarding profession’ (Gupta 2005), whilst today’s economic policies continue to perpetuate this agrarian crisis. For example, The National Skill development policy has detailed/laid out/announced plans to reduce the farming population from 52% to 38% by 2022 (Sen 2019). Thus, farmers continue to cultivate losses.

Indian farmers have become increasingly marginalised in social, economic, cultural and political spheres. They have lost both the capacity to negotiate politically and the ability to be represented within government schemes intended for their benefit (Talule and Rasal 2008). Over the last 18 months, farmer organisations across the country have attempted to come together and exert pressure on the policymakers for agrarian reforms. In 2018, about 200 farmer organisations formed a coalition and staged protest marches in Mumbai and Delhi demanding increased minimum support prices, one-time unconditional loan waiver and importantly, a special session in the parliament to discuss agrarian distress (Agarwal 2018). More recently, over the past 10 months, this protest has grown into mass gatherings and rallies mainly in the States of Punjab, Haryana and western Uttar Pradesh, with over half a million farmers camping at Delhi’s borders. ‘*Since early February, iron nails, rods, barbed wire, boulders and makeshift walls have been used to barricade the capital’s entry points against the protesters’.* (BBC 27 September 2021). This resistance has resulted in several clashes with State police that have frequently led to violence including injuries, suicides and deaths, and storming of the Red Fort in Delhi on India’s Republic Day celebrations. Several nationwide strikes in support of the farmers have so far involved over 250 million Indian citizen participating in solidarity. The farmers have demanded that the State repeal controversial farm legislation passed by the Indian Parliament in September 2020 (Hardikar 2021). Farmer groups have likened these laws as a ‘death penalty’.

Many small and marginal farmers who were affected by agrarian crises resorted to taking their own lives. The suicide rate among Indian farmers is now 47 per cent higher than the national average (Baba 2015; Government of India 2011). Nearly 400,000 farmers have committed suicide in the country between 1995 and 2018 (NCRB 2019). This translates into approximately 48 farmer suicides every day. Published literature consistently suggests that small and marginal farmers are disproportionately affected with the majority farmers belonging to ‘backwarded’ castes (Vasavi 2009; Merriott 2016; Alt 2018) yet the relationship between caste identity of cotton farmers and suicide remains unexamined. A notable exception is research conducted in Maharashtra State which focuses on the voices of widows in the context of patriarchy, caste, and neoliberal agricultural policies (Falnikar and Dutta 2019).

Agriculture in India is embedded in the local culture. Culture in this context is defined as comprising of ‘a cluster of fundamental building blocks of agriculture production processes, rural life and their actors, whose understanding is indispensable for grasping the deeper essence of agriculture’ (Cernea 2005). Culture, in the form of the social organisation, determines the relations, processes, institutions, beliefs, practices and value systems associated with agriculture. Caste-based social organisation form an important component of the village culture. By tradition, caste identity determines the relationship between the people and their land.

## Study area

Ethnographic fieldwork for this study was conducted in a cotton-growing village of the Warangal district, Telangana state. Warangal witnessed a high number of suicides, with more than 500 cotton farmers killing themselves in 1998 and was considered an epicentre for farmer suicides. This tragedy drew international attention from the media, activists and researchers (Herring 2008; Stone 2002). Hasanparthy was one of the Mandals that reported a high number of suicides in Warangal district. Research has highlighted that the majority of farmers who committed suicide during 2004–2010 in the combined state of Andhra Pradesh before Telangana state formation, belonged to Dalit caste communities. Out of the total suicide deaths during this period, 60% of the farmers belonged to ‘backwarded’ castes and 34.4% to scheduled castes (SCs) and Scheduled Tribes (STs)^2^ (Eenadu 2010; Revathi 1998; Sudhakumari 2002).

Over the past four decades, crop patterns in Warangal region changed from coarse food grain to rice and cotton, owing to the production of goods suiting the consumer market (Vakulabharanam 2005). Cotton farming was introduced in the late 1980s in the district (Bharathan 1998). With a sharp rise in the total acreage of cotton in the district, many small and marginal farmers shifted to cotton cultivation. In 1998, severe Bollworm^3^ attacks caused cotton yield to decrease substantially. In response to the American Bollworm infestation, genetically modified Bt cotton was introduced in 2002 by large MNCs and promoted as a panacea for the woes of small farmers. It promised reduction in costs of cultivation, pesticides and higher profits (Qayum and Sakkhari 2005).

The study village is historically dominated by the Reddy^4^ caste group. Power is vested in this group by their caste status, authority, landholding, political power, literacy and connection with urban elites. Most of the bigger landlords belonging to the Reddy caste migrated to cities in pursuit of better opportunities for trade and education. They sold their lands to the villagers. Some of them still own the land, which is cultivated by tenant farmers. The place vacated by the big landlords during the 1980s was populated by entrepreneurs from ‘backwarded’ castes. This led to the rise of small and marginal farmers in the village, largely from the ‘backwarded’ castes and scheduled castes.

## Objectives

1. What is the role of caste in mediating distress amongst marginalised farmers and their families?
2. What are the socio-cultural antecedents that lead to Dalit cotton farmers’ suicides?
3. What are the psycho-social consequences of suicide among their surviving family members?

## Methods

Ethnographic fieldwork was conducted in Hasanparthy Mandal^5^ of Warangal district by the first author (NK) – a medical anthropologist, during 2011–2013. NK’s familiarity with the region and fluency in Telugu were additional reasons for choosing this site for fieldwork. The first author lived at the study site for a year, shadowing farmers in the village and participated in their cotton agricultural activities. As a part of fieldwork, he regularly interacted with key stakeholders in the village from various walks of life, including agriculture and health care.

Five ethnographic case studies of the farmer families are documented and analysed. The case studies selected were based on suicides linked to cotton farming and their Dalit caste background. Interviews with the respondents were conducted at the residence of the families on multiple occasions and at their time of convenience. All interviews were conducted in Telugu, transcribed and *transliterated* into English. Verbatim quotes from subjects, ethnographic observation and field notes supplemented data gathered from interviews. Names of subjects have been changed to ensure confidentiality and to protect individual and family identities.

## Findings

### Case study 1

Mallesham is a thirty-year-old unmarried male farmer belonging to the Madiga caste.^6^ His elder brother Yellaiah, a cotton farmer, committed suicide in 2007. Mallesham and his family live in the SC colony^7^ on the outskirts of the village. He studied until 7th class in the government Telugu^8^ medium school after which he discontinued further studies as this would have meant travelling to Hanamakonda or attend a private school, which the family could not afford. At the time of the interview, Mallesham was viewed as a drunkard (*Tagubothu*) in the village. He would be seen consuming large quantities of alcohol throughout the day and on a regular basis. NK himself observed Mallesham drunk during several meetings with his family.

The interviews took place four years after Yellaiah killed himself. Discussions with Mallesham explored his alcoholism, antecedents of suicides in his family and their perception about caste identity and mental health morbidity.

Under the government land distribution scheme, Mallesham’s father was allocated nearly two acres ^9^ of barren land on the outskirts of the village. Mallesham’s father and his three brothers wanted to shift from their ‘traditional’ occupation - cobblers/leatherwork - perceived to be economically less rewarding. Mallesham’s father also wanted his children to shift from their caste-based occupation, considered to be stigmatising by upper caste communities.

Bt cotton was introduced into their village by a local upper-caste seed dealer and farmer, Srinivas Reddy. This dealer encouraged all farmers to shift to cotton cultivation arguing it would be more profitable. He sold hybrid cotton seeds that promised high yields and floated schemes that offered a rebate for pesticide costs. After three years of a good yield, there were fewer rains, and a new variety of pest attacked the crop. Heavy usage of pesticides did not salvage the crop. By the fourth year, Mallesham’s family was pushed into debt following extensive borrowings from private money lenders. Sensing their distress, an upper-caste farmer offered to pay off Mallesham’s debts in exchange for his land. Faced with this difficult dilemma, Yellaiah (the eldest brother) became deeply distressed and claimed moral responsibility for his family’s predicament. One day, he went to the fields and never returned. Later, it was established that Yellaiah had committed suicide by consuming pesticide ironically bought to save his crop. Within three months, Yellaiah’s wife committed suicide. His elderly father died within six months. Following these multiple bereavements, the entire family became deeply distressed.

Narrative clips from interviews with Mallehsam whilst sober, reveal the complicated relations between caste identity, occupational mobility and mental morbidity. When discussing problems related to caste identity and shift of occupation from cobblers to farmers, he said that:

I don’t think all this would have happened if we belonged to some other caste. My brother wouldn’t have committed suicide. He wanted to see us in a better position by shifting to agriculture, a more respectable work than our caste work, involving leather and dead animals.

The Reddys and other upper castes never endorsed our effort to shift from our caste work to agriculture. They even made fun of it, saying that we low caste fellows know nothing about agriculture.

While explaining problems related to the shift in occupation, including the use of Bt cotton and access to natural resources and debts:

My father was not willing, but because of the government land allotment, my brothers became ambitious and wanted to venture into agriculture. We started growing paddy and shifted to cotton cultivation as everyone else in the village was doing.

Water was always a problem as we had to pump it from the canal through the lands of the big farmer from the Reddy caste [who controlled the amount and timing of water supply]….when the crops failed only the private money lender was accessible and ready to give us loans. We know the interest rates were high, but there was no other source.

Finally, when drawing the link between his brother’s suicide, deaths in his family and his alcoholism:

Our loans mounted and my brother started blaming himself for the situation. One day he went to the fields and never came back. His body was found in the fields the next day. He died after consuming pesticides. My father, who was old, could not bear this and he died heartbrokenly. My brother’s wife committed suicide by consumed pesticide.

I don’t do anything. I sometimes drink ‘mandu’^10^ as it gives me relief. If we were from any other caste we could have got loans and support, and we wouldn’t have been in this situation.

### Case study 2

Srinu is a thirty-two-year-old male farmer belonging to the Mudiraju caste.^11^ His elder brother Kumar, a cotton farmer, committed suicide in 2010. Fishing was the traditional occupation of Mudiraju community in the village. Until a couple of decades ago, his community were primarily dependent on fishing for their livelihood.

The deceased farmer Kumar, started to cultivate Bt cotton in 2008. The family owned 2 acres of land. Kumar leased three additional acres adjacent to his land in tenancy from an upper caste family in the village. Though the returns were not as high as expected, he continued investing in cotton cultivation by borrowing money from his relatives and villagers. His expectation of a good harvest and remunerable price were not fulfilled for two years in a row. He was under increasing pressure from the money lenders to return the money he borrowed together with interest, yet he was unable to pay the agreed tenancy amount for the extra land. Kumar was upset when one of the money lenders came to his home and demanded repayment in front of his wife and family. He committed suicide less than a week later. During the interview, his younger brother Srinu mentioned that Kumar had felt humiliated and unable to interact with family members after the incident.

While discussing their shift of occupation from fishing to cotton farming, Srinu commented:

Traditionally we used to raise fish in the village tanks … that was our primary activity [and] agriculture was mostly secondary … as there were less rains … there aren’t many fishes … unlike earlier years fish do not grow and weigh less in the lakes in the village … most of us do not know anything else….

We do not have any option available other than agriculture. We are not skilled or trained to doing anything else. Agriculture is the only option. Either we float or sink.

Later, when exploring the role of Bt cotton, market rates and his brother’s tenancy, he said:

We took up cotton cultivation in small bits of land we have and took tenancy … we could not do anything else as agriculture is a full-time activity.

Some years rates were good … and some people made some profits … seeing them many of us started cultivating cotton … either the yield is more and priceless or the price is more, and we do not have much yield.

Tenant farming is a waste … my brother lost all his investment in tenancy … input costs have grown so much, but prices remain low … small farmers cannot develop doing agriculture. They can barely survive.

Kumar’s widow, Rani, and Srinu were asked to narrate the antecedents of suicide and its impact on the family. When reasons for his brother’s suicide were explored, they stated that:

People who do not care about what others say will live … People with little Izzat (honour). Once the moneylender pressurises for repayment, they lose their face amongst the community… this they cannot tolerate, and they commit suicide.

This happened in the case of my brother. He felt humiliated^12^ and could not face all of us. Many of us are in a similar situation.

Srinu currently cultivates cotton and maize. He thinks maize is a better option than cotton as the input costs are relatively fewer. However, he continues to cultivate cotton, hoping for better returns.

Rani recollected that despite her apprehensions, her husband Kumar was keen to take up land for tenancy. She mentioned that he began drinking alcohol during this phase, but never expressed the wish to end his life. She also expressed concern about her 10-year-old son, who had become socially withdrawn after his father’s death.

When Rani was asked to narrate the antecedents of her husband’s suicide, she said:

He took the land for tenancy and invested heavily on cotton. He loaned about one lakh rupees (1,600 USD) from village people only. He could not repay as cotton crop failed consecutively for two years. People who lent him money started to put pressure on him. He could not tolerate that and consumed pesticide and died.

He started to drink mandu more than his usual before he killed himself. But he never ever showed a sign that he will commit suicide.

While discussing the impact of suicide on her and their children:

I have repaid some part of the loans. Now [there are] fifty thousand more (800 USD) … how can a woman repay so much money?

My elder son does not study well … he is mostly lost … mostly silent … will not listen to us … He does not speak much. He was close to his father. He is not studying. He just stares at someone if they speak to him. I do not know what to do with him.

Rani and her children live nearby in a house bequeathed to her by the parents of her deceased husband. She works as farm labour during the cotton-picking season in order to make a living. She also tends to an extended kitchen garden to grow vegetables and sells them to a village vendor.

### Case study 3

Pramila is a thirty-year-old woman whose husband committed suicide in 2010. She has two young children aged seven and five. Her husband, a cotton farmer, committed suicide after consuming pesticide following consecutive years of crop failure. They too belong to the Mudiraj community – traditionally a fishing caste occupation.

Over the course of several interviews with Pramila, NK discussed with her the following issues: her husband’s suicide, family’s credit sources, the productivity of cotton, her own health condition, and help-seeking.

Pramila’s husband owned two acres of land, which he inherited from his father. His father used to grow paddy, vegetables and a small amount of cotton. Her husband shifted from growing vegetables to Bt cotton after watching his friends and fellow farmers doing well. He borrowed money for initial investment from a private money lender, and though the crop yield was good in the first year, the returns were not enough to clear his debts. He continued farming cotton, and over four years, the cotton yield decreased. He was forced to buy more fertilisers hoping that it would help him get better yields. Over time, he came to owe money to both the local village seed and the pesticide dealer. Mounting debts and decreasing returns from cotton pushed him to commit suicide. His wife mentioned that he was a kind and sensitive person and never expressed frustration to her or their children.

A few months after her husband’s suicide, Pramila started to experience fatigue and chills in her body, together with suicidal ideation. She could not go to work because of her condition. There was pressure on her to repay the loans, complete the construction of her house and support the education of her children. She felt that her husband’s family wanted to take possession of the house which her husband had started to construct. She now works as an agricultural labourer for her living.

When asked to explain the genesis of her health condition, she said:

My problems started after my husband died. I never had any problems before then. I suddenly started feeling that something is in my head and it is putting bad thoughts into my head. I felt like dying. But I restrained myself because of my two small children … their father is gone if I too die who will take care of them? … My house and everything will be snatched away. My parents advised me to visit them. I did not want to leave the house and go. But my condition worsened and I did not know what I was doing. A couple of times I had fits and it seems I was shouting that I wanted to die and this was unstoppable. It took three people to hold me down.

When asked about seeking help for her illness and impact on her family, she said:

After all this, my parents came and they took me to a lady who offers cures for such illnesses.^13^ All the villagers call her “attamma” (aunt). She performed some rituals and told me that someone had cast a spell on me. We sacrificed a chicken and performed some rituals as suggested by her. I was OK until sometime after this. Then after a while, my situation got worse. I was always under the impression that men around me were eyeing me with bad intentions. Those thoughts always crept in my mind. I went to the village Hanuman temple (Monkey God) and did puja (offered prayers) as suggested by the pujari (priest). Later, after somebody’s advice, I went to a Darga (Muslim shrine) near my parents’ village. There they gave me something to drink and I vomited. A black pellet was found in my vomit and it had some rice and dal remnants in it … The Darga seer said that someone had cast a spell on me through food and I would now be OK. He gave me an amulet. All this was financially very draining. I spent so much money, but things did not improve. After the Darga visit, I was feeling better but still had bad thoughts coming into my mind some times. Later my parents took me to a mind doctor (psychiatrist) in the town nearby to my paternal village. He asked me everything and said I have some problem. He gave some medicines for one month. I am better, but the heaviness in my head continues. My children are suffering. I am worried about my elder son. He was already disturbed by his father death. And my condition is affecting him more.

Pramila was taking her medicines (two anti-depressants and a benzodiazepine) prescribed by the psychiatrist when last interviewed. She also continued to visit a local temple and seek the advice of the temple priest.

### Case study 4

Rajender is a twenty-eight year old cotton farmer. His father, also a cotton farmer, committed suicide in 2001. He belongs to the Kuruma caste.^14^ Traditionally, they are shepherds who tend sheep and make woollen blankets. Many families over the last decade gave up sheep rearing. Grazing sheep was increasingly difficult as most of the lands in and around the study village were converted into agricultural lands. Some Kuruma families in the village-owned small bits of land in which they had grown paddy, vegetables and cotton. There was a demand for meat but there was no demand for the woollen blanket (*gongadi*) which they wove. Consequently, most of the Kuruma families in the village shifted to agriculture as a primary occupation and grew cotton.

During the interviews with Rajender, discussions centred on the difficulties of shifting occupation, growing commercial crops like maize and cotton, the impact of his father’s suicide on his family, his alcoholism, his mother illness, and his own attempt to commit suicide.

While discussing antecedents to his father’s suicide, he said:

My grandfather had a big herd of sheep. He bought three acres of land. After he passed away, my father was not keen on tending sheep, and he sold them off. He started cultivation of maize and cotton, seeing that everyone in the village had done the same. Later he shifted completely to cotton.

This was before Bt cotton was introduced in the village, but now most of the cotton farmers in the village grow hybrid cotton. He planted hybrid cotton in our land and also took two acres of land for tenancy. During a particular season, cotton was heavily infested with American bollworm and this lead to heavy damage.

He borrowed fifty thousand rupees (800 USD) from our relatives. He did not want to go to others. My relatives gave him a loan as we had land. He went on paying interest on interest as cotton crop failed in consecutive years. He was very upset that he was unable to support the family despite all the hardships.

Relatives who gave loans to him started demanding the money. A couple of them asked him about it a few times. He had never faced such a situation in his life. We can never tolerate somebody mocking us. He felt humiliated. One day he went to his father’s grave near our field and consumed pesticide and died.

Commenting on his mother’s illness, he said:

Soon after hearing about my father’s suicide, my mother fainted and fell down. She started to have bouts of severe headaches since then. She complains about a numbing sensation in her head, dizziness. With this condition, she is unable to go to our field to work. We are forced to pay for extra labour. We have taken her to good doctors and spent a lot of money. Recently, we went to a big hospital, and they scanned her head. All of them say there is no problem. The doctor gave her some medicine. She does not take the medicine regularly. She forgets and takes it whenever she remembers. We are at a loss and do not know what to do. I think my father’s death has got into her head and affected her.

On questions about his own life he said:

I stopped my education after the 9th class because I had to work to support my family. I have learnt about driving, and can drive heavy vehicles and auto.^15^ I was working as a tractor driver in the village. My brother is studying in Hyderabad. I support him. He wants to go for higher studies and all want him to study and become something. I got married to my maternal cousin. I was managing the farm and started to drive an auto. Somehow I got into the habit of drinking. It got so bad that I was driving the auto mostly drunk. One day I was driving the auto with passengers and met with an accident. I broke my leg. The passengers escaped with few injuries but I was hospitalised. Most of the villagers looked down upon me. I felt humiliated and thought I should die. I consumed pesticide meant for cotton. My wife spotted me, and villagers rushed me to the local government district hospital immediately and I survived. I am unable to completely give up alcohol.

When NK contacted him last in 2013, Rajender was taking care of his mother. He cultivates cotton and maize in their field. He was trying for a job as a driver in the government transport department, and trying hard to give up alcohol.

### Case study 5

Jyothi is a twenty-four year- old woman whose husband Ramesh, a cotton farmer committed suicide in February 2013. They have a six-year-old daughter. They belong to the Kummari community,^16^ who traditionally made earthen pottery.

Two decades ago, some Kummari families in the village bought lands from the family of Dora, the village landlord belonging to the Reddy caste. Dora and his family owned most of the lands in the village. As the land was more fertile and had water sources for irrigation, most of the Kummari families prospered and bought more land from others in that area. Most farmers from this community were hardworking and grew vegetables, fruits and paddy along with cotton. Some small farmers who had less land shifted to growing cotton.

Discussion during the interviews with her centered largely around cotton cultivation, sources of credit, antecedents of her husband’s suicide and its impact on his family.

While discussing occupation shift and agriculture, she said:

Traditionally we belonged to a community of potters. But we left that occupation some decades ago as making pots was not profitable. My father-in-law brought one and a half acres of land and started cultivation. He was able to support the family’s needs with agriculture. My husband who inherited the land started to grow paddy and chillies as we have a well as a source of water.

When discussing cotton cultivation and causes of debts she said:

A few years ago, as everyone was planting cotton, we also planted cotton. Since the last few years, the yield from cotton has been minimal. We did not make any money out of cotton. This year too we have borrowed money. Our debts are about ninety thousand rupees (1440 USD).

Our crop was destroyed because of heavy rains. My husband tried to borrow from relatives to repay the loans but was unable to raise enough money. My brother who loaned us money said he could support us but my husband was not willing to accept his help as we already owed him. Though the government officials visited and promised compensation for lost crops, my husband was not convinced that it would help him repay the loans.

During his last days he was always worried about money. We could not do anything but suffer. When we went to attend a marriage of our relatives, one who loaned us money asked us about repayment. All the men were drinking … after returning home, he went to the fields and consumed pesticide and died. We never thought he would do this … he did not talk to any of us about taking his life.

I don’t know what to do now. I am worried about the future of my daughter and myself. I will have to live like this. I hardly get sleep, often I for many nights at a time. I cry and am nervous all the time about the future. My daughter is too young to understand, but she will not have a father. I am worried how I will take care of her.

When NK last interviewed Jyothi in January 2013, she was yet to come to terms with the loss of her husband. She felt unwell as she had problems with her sleep and appetite. She was awaiting compensation from the government. A leader from a regional political party in the opposition has donated some money to her family.

## Discussion

The case studies presented do not suggest a direct causal link between caste identity distress, cotton farming and suicide. Instead, they highlight the complex interplay of various factors leading to suicide among Dalit cotton farmers.

Case study 1 illustrates the distress caused in Mallesham’s family because of their attempt to move up the social order. Their problems included access to land and natural resources, access to education, the ability to generate capital and lack of social support. Structural and social discrimination was evident in the family’s inability to generate capital. Mallesham and his family aspired for a respectable occupation. This case study demonstrated that they could not move beyond the boundaries of the caste system. The humiliation and bereavement experienced by the family resulted in three deaths. This pushed Mallesham to a state of despondency resulting in a dependence on alcohol.

In case study 2, Kumar killed himself as the expectations of his cotton crop and its returns were not met. His inability to repay his loans led him to feel humiliated by the lenders. This case study highlights yet again the relations between caste-based occupational mobility, notions of honour and humiliation, alcohol dependency, suicide and severe mental illness.

All of the case studies suggest that the social suffering of the farmers is mediated by the cotton crop. The social suffering experienced by the families of the farmers who committed suicide was embodied in their patterns of distress. In case study 3, for example, Pramila never had any health problems until her husband died. The distress caused by her husband’s death and continuing demands from her husband’s family to possess her house and land caused her to develop a depressive illness.

This case also highlights local ideas about mental health, health-seeking behaviour amongst the community and the lack of local mental health care services. As the district hospital and private health services were easily accessible, primary health services provided by the sub-centre in the village were unutilised. There was also a general lack of awareness among the villagers regarding mental health services.

Similarly, case studies 4 and 5 indicate the somatisation of stress experienced by the family members. In case study 4, the wife of the deceased farmer has unexplained headaches and dizziness, alongside episodes of dissociation which commenced after her husband’s death. Her son became dependant on alcohol to cope with the loss of his father. He survived a suicide attempt but did not know where to seek professional help. In case study 5, Jyothi was unable to cope with the loss of her husband. She developed sleep and appetite difficulties, and anxiety over her daughter’s future wellbeing - together with the vulnerability to social and physical exploitation of a young female family of two.

Lived experiences in the context of local socio-cultural settings shape the experience of humiliation in the documented case studies. Humiliation is a common theme that cuts across all the five case studies as an important affect leading to Dalit farmer suicides. Although the dynamic of humiliation in western societies has been extensively documented (Smith 2008; Kaufmann et al. 2011), there is little theoretical work to address the phenomenology of humiliation in non-western societies including India (Guru 2011). The case studies presented above suggest the scope for further scholarship in humiliation studies within the Indian context. For example, what factors such as caste identity, State denial, a liberal market economy, and mental health policy constitute and condense into a unique and distressing locally configured *panopticon*? The authors argue that Dalit cotton farmers experience humiliation with little room for manoeuvre. Suicide, therefore, resolves their humiliation and is a logical conclusion to social exclusion that results in the annihilation of their existence.

### Cultivating distress

Cotton was promoted as a panacea for the problems of the rural community. Legitimisation by the State (Menon 2006) and aggressive market strategies of the multinational seed companies (Sainath 2012) led people to believe that cotton farming could change their lives. Communities who took up farming as an occupation considered cotton as an opportunity to improve their social and economic status. Most large farmers who could withstand the vagaries of the produce and the prices have been benefited in the long run, but cotton has been a nemesis for most of the small farmers, accentuating their social suffering along with that of their families.

Non-farming caste groups require cultural, social, economic and symbolic capital to initiate and sustain farming as a profession. It could be argued that social capital is germane to understanding the hardships and mental health morbidity that impacted on these families. Meanwhile, being to a ‘lowered’ position within the caste hierarchy, Dalits and ‘backwarded’ castes lack symbolic capital available on the basis of a person’s position, honour or prestige, which functions as an authoritative embodiment of cultural values.

The case studies also demonstrate that the paucity of social, cultural and economic capital had an adverse impact on the farmers’ lives, leading to excessive social stress. Individuals’ psychosocial contexts and stressors, such as financial hardships, poor education and unfulfilled expectations at work, have been identified as the most common correlates of suicide (Kuruvilla and Jacob 2007). Throughout the data from this research highlights the discrimination faced at the individual, familial and structural levels (Farmer 1997) leading to their marginalisation.

The links between social marginality and mental health in India need to be understood within the framework of the caste system, which enforces stratification and social restrictions. Surprisingly, there are no textbooks, research, nor major international reports which allude to the mental health dimensions of India’s caste system. Exceptions to these are personal Dalit biographies grounded in literary studies (Dangle 2010; Freeman 1980; Hazari 1951; Jiloah 1995; Kale 2003; Limbale 2007; Moon 2002; Sattanathan 2007), theological perspectives (Webster 2007); an interview (Jadhav 1998), interdisciplinary seminars on caste and mental health (Anand 2003; Jadhav, Mosse, and Dostaler 2016), and recent initiatives on caste, stigma and wellbeing including caste mediated distress in higher education (Jadhav 2016; Jadhav et al. 2012). The overwhelming literature focuses on suffering within Dalit communities in India, excluding factors brought about by social and cultural institutions that embody, perpetuate and amplify caste hierarchy (Sunil Menon 2021).

Based on the scarce research conducted on the association between caste and mental health, it is evident that the Hindu caste system enforces a chronic sense of inferiority, inadequacy and insecurity, which blocks the universal urge for self-expression and dignity. This leads to rage and anger manifested in actions against the perpetrators, or directed at oneself (Heinrich 1937). Similarly, cultural psychoanalytical approaches argue in favour of developmental theory to unpack such affective insults. Such theories suggest that family and social hierarchy are internalised during early childhood leading to culturally designated caste roles in life later (Kakar 1983). This argument is elaborated further in literature as ‘introjections of dirt, darkness, pollution and associated fantasies’ (Kakar and Kakar 2011).

A recent study on the Human-Elephant conflict and its mental health consequences propose that asymmetric interactions between people and institutions generate landscapes that are actively ‘counter therapeutic’ (Jadhav and Barua 2012). From the case studies presented in this paper, the authors argue that existing marginalisation of the farmers belonging to ‘lowered’ castes is accentuated by shifting to cotton cultivation. The authors propose that State promotion of cotton crop and open market polices have generated what might be termed as ‘toxic landscapes’ (Kannuri and Jadhav 2018). These result in widening inequity by producing financial, social, cultural and psychiatric distress for cotton farmers and their families. Cotton cultivation literally leads to toxic landscapes as it involves extensive use of fertilisers and pesticides. Though cotton cultivation amounts to only 7% of total crop cultivation in the country, it accounts for 54% of the total annual pesticide and 5% of fertiliser usage. Introduction of genetically engineered Bt cotton adds a new dimension to this toxicity because of concerns relating to genetic contamination. This concept of toxic landscapes has been further elaborated in an earlier paper that explicates how toxicity is generated through a mutually constituted set of institutions including State policies, free-market economies, health care institutions, and cotton farmers (Kannuri and Jadhav 2018). The authors further hypothesise that the current corona virus pandemic and counter measures adopted by the State will generate more social inequalities and disproportionately impact small and marginal farmers with an accumulated history of enforced cultural distancing. This is supported by more recent evidence documented in a rich ethnographic account of Dalit farmers who have survived suicidal attempts and continue to offer resistance (Hardikar 2021).

## Conclusion

This paper addresses and highlights a complex interplay of mental distress shaped by agricultural policies, cultural identities and social emotions. These intertwined factors contribute to cotton farmer suicides together with psychiatric morbidity amongst survivors. In addition, increased toxicity of landscapes resulting from a shift to commercial crops leads to a negative impact on the wellbeing of cotton farmers and their families. This further accentuates existing social inequalities resulting in toxic landscapes that are actively counter therapeutic and lead to the loss of life. This pervasive humiliation and lack of hope is ‘cultivated’ by a cascade of decisions taken by others with little or no responsibility to the farmers and the land they hope to cultivate. Suicide resolves the farmers’ humiliation and is a logical conclusion to farmers’ exclusion from decisions which increase their debt and reconfigure agricultural spaces into socially toxic places, in turn framing a local *panopticon*. This panopticon is transformed and inscribed into registers of local bureaucratic activities including agricultural, finance and health professional records (Badami 2014, 99). The authors of this paper hypothesise that this reframing of the Foucaldian ‘panopticon’ into a crude and potent ‘*panlexicon*’ subjects the farmer to the violent gaze of State biopower. Through reversing this gaze, the suicidal act offers a counter-narrative.

Recent debates in the field of political sociology and medical anthropology suggests that focusing on causal explanation of farmer suicides argue against the interpretation of such tragic deaths as mere resistance to biopolitics and it’s role in governing of life (Alt 2018). Biopower, if understood as the social political control over people’s lives leading to a State sponsored death which in turn, normalises population control, does not in itself explain how such power dictates those who live and those who die. This contestation of Foucauldian biopower has been clearly articlated by Achille Mbembe, Cameroon philosopher, in his seminal essay on Necropolitics: ‘*but under what practical conditions is the right to kill, to allow to live, or to expose to death exercised? Who is the subject of this right? What does the implementation of such a right tell us about the person who is thus put to death and about the relation of enmity that sets that person against his or her murderer? Is the notion of biopower sufficient to account for the contemporary ways in which the political, under the guise of war, of resistance, or of the fight against terror, makes the murder of the enemy its primary and absolute objective?’ *(Mbembe 2003). Alt (2018) however argues that ‘*such theorization fails to recognise both the particularities of biopolitics in a context where the presence of death is ubiquitous and the way in which the death of some may reinforce the biopolitical governance of life of others’ *(ibid. p1). The case studies discussed in this paper do not lend themselves to elaborate on Alt’s interrogation of biopolitical power. However, Alt’s persuasivel thesis poses questions that would further develop an inter-disciplinary research collaboration in order to blur the prevailing problematic dichotomy which either pathologise farmers’ distress as mental disorder or focus on external social, economic and political explanations.

This study argues that further research on a larger scale in regions reporting suicides amongst cotton farmers to elicit local cultural factors is both crucial and urgent to prevent the loss of large number of members from India’s farming community. This is particularly relevant at a time when national and international concerns over food security are being actively debated by politicians, economists and social scientists (FAO, IFAD, UNICEF, WFP, and WHO 2021).

To proceed further requires culturally sensitive health care systems that involve active co-ordination and dialogue among cotton farmers, local agricultural and mental health policy makers, clinicians, social scientists, and public health professionals. Space limits detailing further steps for each of these disciplines. For example, cultural sensitivity of public health professionals could be enhanced with an orientation in clinical anthropology.^17^ Without such work, clinical training and health systems in India will continue to marginalise locally relevant socio-cultural aspects of health problems faced by a majority rural population who place their trust in India’s official bio-medical network (Jain and Jadhav 2008; 2009; Barua et al. 2021). Such an approach offers potential to generate local culturally valid theory in mental health including the central role of caste identity distress among marginalised communities (Jadhav 2009).

The limitations of this study include the purposive selection of subjects among cotton farmers belonging to Dalit castes, although they represent the majority of suicides amongst cotton farmers in the study site. The paper does not address how upper-caste cotton farmers cope with crop failures nor does it enquire into the lives of Dalit famers who continue to endure the ongoing agrarian crisis.

## Ethical approval

Ethics permission was provided by UCL ethics committee, project ID number 2846/001 and IIPH Hyderabad, PHFI India ethics committee.

## Notes

1 A marginal farmer is a farmer who cultivates agricultural land up to 1 hectare (2.5 acres) and a small farmer is one who cultivates land of more than 1 hectare to 2 hectares (5 acres) (Mishra et al. 2006).

2 Indian constitution categorised historically disadvantaged communities according to their position in the caste system as a criterion for providing social and developmental benefits equivalent to positive discrimination. Accordingly, most of the castes belonging to shudra varna are classified as ‘‘backward’’ castes and those belonging to ‘untouchable’ communities are classified as scheduled castes and *Dalits* (the latter is a self ascribed term meaning ‘oppressed’). for generations. The term ‘‘backward’’ was first used in 1884 by British colonial officials’ proceedings to denote people who were socially and economically marginalised. The association with Race in the original source makes its eugenic connotation explicit. This term has since been enshrined in the Indian Constitution and continues to be used in official Indian State reports and Indian media without its association with Race. See 1884 Rep. Director Public Instr. Bombay Presidency 1883–4 App. L. p. xcix [Recommendation] No. 12 recommends larger grants in ‘backward’ districts and for ‘backward’ castes and races. Oxford English Dictionary. Online edition. 1991. https://www.oed.com/. However, the usage of the terms ‘backward’/’lower’ castes is contentious as they signify historical oppression and unequal social recognistion that is embedded in the official and social languages (Mosse 2018). The authors have therefore deliberately changed the terms ‘backward’ and ‘lower’ to the verb form, ‘backwarded’ and ‘lowered’ wherever appropriate so that they help the reader identify social vectors that frame their identity and experience. For more details on de-dominalization and verbing, see Clark and Clark 1979.

3 Bolloworm (Helicoverpa armigera) is a moth which infests cotton. The larvae attack blooms, bolls and growing tips of the cotton plants causing heavy losses.

4 The Reddys are one of the powerful landlord caste groups in Andhra Pradesh. Historically, they were rulers between 1353 to 1448CE. Later they continued to be chieftains, village heads, village policemen, tax collectors and farmers in the Telangana region. Reddys dominate modern day politics in Andhra Pradesh as a majority of chief ministers and cabinet ministers over a period of time belong to this caste.

5 Mandal is an administrative unit at the sub-district level.

6 The traditional occupation of the Madigas is leather work (cobblers). The Hindu caste system places the Madigas out of the Varna system. They are considered as ‘untouchables’ based on the principle of ritual purity and impurity. According to the Indian constitution, the Madigas are categorised as a scheduled caste (SC). Currently, the former untouchable caste groups prefer to call themselves as Dalits.

7 In most Indian villages, housing pattern is based on caste based segregation. The upper castes dwell in the centre and the scheduled castes reside on the outskirts of the village. The Government Housing scheme meant for constructing pucca houses for Dalits conform to this segregation and commonly termed as SC colony.

8 The local language spoken in Andhra Pradesh.

9 1 acre = 43560 square feet.

10 Mandu is a local term of liquor. This term is synonymously used for medicines and pesticides. Most of the villagers consume toddy, an extract from borassus palm tree; locally brewed illicit liquor and Indian made foreign liquor. For toddy, most men go to the nearby toddy groves. Locally brewed country made liquor is sold in sachets in a shack hotel farther from the village. The village also has a small liquor outlet commonly known as “belt shops” to sell a range of cheap liquors.

11 According to Hindu caste system, Mudiraju community are classified as Shudras, the fourth category of the Varna system. Indian constitution categorises Mudiraju as a ‘backward’ caste (BC).

12 The common phrases denoting humiliation in Telugu are: Izzat teesindru, siggu poyindi, paruvu teesindru.

13 This lady is a traditional healer, locally called as Mantrala Attamma. She gets possessed and diagnoses the cause of the suffering of clients who visit her. Most of these traditional healers offer to explain their clients predicament deploying supra natural categories. Their intervention is usually in the form of offering sacrifices to propitiate the local goddesses.

14 According to Hindu caste system, Kuruma community is classified as Shudras, the fourth category of the varna system and a ‘backward’ caste (BC) community group according to the Indian constitution.

15 Auto is a three wheeler motor vehicle commonly hired as a means of transport

16 According to Hindu caste system, Kummari community is classified as Shudras, the fourth category of the varna system and a ‘backward’ caste (BC) community group according to the Indian constitution.

17 Clinical anthropology is a short term for anthropology in clinical medical settings, or anthropology related to clinical medical activities, including the preventive orientation of public health and the primary care orientation of community health (Maretzki 1980).
